# Applications, Challenges, and Prospects of Generative Artificial Intelligence Empowering Medical Education: Scoping Review

**DOI:** 10.2196/71125

**Published:** 2025-10-23

**Authors:** Yuhang Lin, Zhiheng Luo, Zicheng Ye, Nuoxi Zhong, Lijian Zhao, Long Zhang, Xiaolan Li, Zetao Chen, Yijia Chen

**Affiliations:** 1 Guangdong Provincial Key Laboratory of Stomatology, Hospital of Stomatology, Guanghua School of Stomatology, Sun Yat-sen University, No. 56, Lingyuan Road West, Guangzhou, 510055, China, 86 13580591020; 2 Zhongshan School of Medicine, Sun Yat-sen University, Guangzhou, China; 3 School of Government, Sun Yat-sen University, Guangzhou, China

**Keywords:** generative artificial intelligence, GAI, large language model, ChatGPT, medical education, human-machine symbiosis

## Abstract

**Background:**

Nowadays, generative artificial intelligence (GAI) drives medical education toward enhanced intelligence, personalization, and interactivity. With its vast generative abilities and diverse applications, GAI redefines how educational resources are accessed, teaching methods are implemented, and assessments are conducted.

**Objective:**

This study aimed to review the current applications of GAI in medical education; analyze its opportunities and challenges; identify its strengths and potential issues in educational methods, assessments, and resources; and capture GAI’s rapid evolution and multidimensional applications in medical education, thereby providing a theoretical foundation for future practice.

**Methods:**

This scoping review used PubMed, Web of Science, and Scopus to analyze literature from January 2023 to October 2024, focusing on GAI applications in medical education. Following PRISMA-ScR (Preferred Reporting Items for Systematic Reviews and Meta-Analyses Extension for Scoping Reviews) guidelines, 5991 articles were retrieved, with 1304 duplicates removed. The 2-stage screening (title or abstract and full-text review) excluded 4564 articles and a supplementary search included 8 articles, yielding 131 studies for final synthesis. We included (1) studies addressing GAI’s applications, challenges, or future directions in medical education, (2) empirical research, systematic reviews, and meta-analyses, and (3) English-language articles. We excluded commentaries, editorials, viewpoints, perspectives, short reports, or communications with low levels of evidence, non-GAI technologies, and studies centered on other fields of medical education (eg, nursing). We integrated quantitative analysis of publication trends and Human Development Index (HDI) with thematic analysis of applications, technical limitations, and ethical implications.

**Results:**

Analysis of 131 articles revealed that 74.0% (n=97) originated from countries or regions with very high HDI, with the United States contributing the most (n=33); 14.5% (n=19) were from high HDI countries, 5.3% (n=7) from medium HDI countries, and 2.2% (n=3) from low HDI countries, with 3.8% (n=5) involving cross-HDI collaborations. ChatGPT was the most studied GAI model (n=119), followed by Gemini (n=22), Copilot (n=11), Claude (n=6), and LLaMA (n=4). Thematic analysis indicated that GAI applications in medical education mainly embody the diversification of educational methods, scientific evaluation of educational assessments, and dynamic optimization of educational resources. However, it also highlighted current limitations and potential future challenges, including insufficient scene adaptability, data quality and information bias, overreliance, and ethical controversies.

**Conclusion:**

GAI application in medical education exhibits significant regional disparities in development, and model research statistics reflect researchers’ certain usage preferences. GAI holds potential for empowering medical education, but widespread adoption requires overcoming complex technical and ethical challenges. Grounded in symbiotic agency theory, we advocate establishing the resource-method-assessment tripartite model, developing specialized models and constructing an integrated system of general large language models incorporating specialized ones, promoting resource sharing, refining ethical governance, and building an educational ecosystem fostering human-machine symbiosis, enabling deep tech-humanism integration and advancing medical education toward greater efficiency and human-centeredness.

## Introduction

### Background

The 21st century has seen accelerated advancement in information technology and artificial intelligence (AI), significantly altering lifestyles and work paradigms. With progress in deep learning and large-scale data processing, generative artificial intelligence (GAI) has emerged as an influential innovation. GAI rapidly expands into diverse applications, enabling content generation across text, images, and audio through the analysis of extensive datasets [1]. Its market demonstrates notable growth, with a 2024 global valuation of ~US $16.8 billion and a projected 37.6% compound annual growth rate (CAGR) from 2025 to 2030 [2], reflecting its significance in commercial and academic domains.

GAI’s development is driven by advances in natural language processing (NLP), particularly the Transformer architecture, which enables the generation of complex content. Large language models (LLMs) serve as core technical implementations of GAI. Models like GPT-3, GPT-4, Copilot, and LLaMA 3 have expanded GAI applications from basic automation to sophisticated tasks including content creation, data analysis, and intelligent question-answering systems [3]. These transformer-based LLMs exemplify how conceptual GAI frameworks are operationalized via model architectures and engineering practices.

With technological advancements, GAI has gradually infiltrated more specialized fields, with medical education a prime example. This domain faces challenges due to its knowledge-intensive and highly practical characteristics: traditional teaching methods struggle to replicate clinical scenarios efficiently, and increasingly scarce clinical teaching specimens and patient resources limit the clinical practice training of medical students, all of which are not conducive to the cultivation of medical talents with both clinical thinking and practical ability [4]. In this content, GAI may empower medical education through its enhancement effects on 3 core educational elements: improving resource generation efficiency, optimizing the interactivity of pedagogical approaches, and enhancing the automation level of assessment processes [5,6]. Nevertheless, the accompanying integration risks include potential biases and inaccuracies in generated content [7] and possible inhibition of critical thinking through over-reliance [8]. Thus, optimal implementation strategies warrant further investigation.

Current GAI integration in medical education involves rapid technological iteration and shifting research paradigms [1,9,10]. Prior reviews exhibit three limitations: (L1) Overreliance on single-model analyses (predominantly ChatGPT) [9,10], (L2) insufficient examination of geographical disparities in adoption patterns, and (L3) fragmented assessment of GAI’s impact across 3 core dimensions of medical education. These dimensions include resources (teaching support materials like GAI-generated clinical cases and pathological images), methods (instructional strategies like adaptive learning pathways and simulated decision-making), and assessment (automated evaluation of learner performance, such as automated short-answer scoring). Crucially, studies before 2023 were constrained by the technology’s maturity, missing the recent shift from theoretical exploration to operational implementation [1]. Therefore, a new round of scoping review is urgently needed to focus on the critical evolution period between January 2023 and October, 2024 (before the completion of this scoping review), construct a multidimensional analytical framework (encompassing resources, methods, and assessment), and clarify the complex picture of the deep interaction between GAI and medical education. To guide this investigation, this study discusses the multifaceted landscape of GAI adoption in medical education through 3 interconnected lines of inquiry. First, it aims to examine whether regional disparities exist in GAI implementation and how researchers exhibit preferences for specific LLMs (eg, ChatGPT). We posit that adoption patterns will demonstrate significant stratification aligned with national development levels and reflect preferential usage of widely accessible general-purpose models. Second, it seeks to map the current state of GAI applications across educational resources, methods, and assessment dimensions. We hypothesize that effectiveness will vary substantially across these domains due to differences in technical implementation requirements and inherent task complexities. Third, it intends to identify current limitations and future challenges, positing that technical deficiencies, including ethical risks such as compromised academic integrity and data hallucinations, will constitute the most significant barriers to sustainable integration.

### Theoretical Framework

#### Theoretical Model: The Theory of Symbiotic Agency

Based on the theoretical framework of symbiotic agency [11], a theory emphasizing interdependent and collaborative relationships between humans and technology, this study conceptualizes human-technology relations as a process of mutual constitution. Technology functions neither as a passive instrument dominated by humans nor as an autonomous replacement for human agency. Instead, it develops in tandem with humans through interdependent interactions: technology enhances human efficacy by expanding cognitive boundaries and enabling novel multimodal interactions, while humans legitimize technological practice by embedding ethical norms and conducting context-specific interpretations such as weighting clinical decisions. This symbiosis transcends traditional master-servant dichotomies by establishing a responsibility-sharing network. Within this network, technology acts as a co-agent in human activity systems, collectively enhancing capabilities rather than substituting human roles. This perspective provides the foundational understanding needed to maintain a dynamic balance in human-technology interdependence within medical education, forming the basis of our conceptual model.

#### Conceptual Model 1: Specialized Models Integrated System Based on General Large Language Models

Building upon the analytical framework established in Table S1 in Multimedia Appendix 1, which systematically compares general-purpose and domain-specialized GAI models across 3 critical dimensions (knowledge representation fidelity, task compatibility, and ethical constraint mechanisms), this study deconstructs technological heterogeneity to avoid conflating “GAI” as a homogeneous entity. The models in Multimedia Appendix 1 (see Table S1) were selected via multisource evidence synthesis, including peer-reviewed studies, industry reports (eg, Global Large Language Model (LLM) Market Research Report 2024) [12-20], and empirical validation in educational contexts, based on four criteria: (1) technological representativeness of core advancements (multimodality, reasoning, and domain adaptation); (2) broad academic and practical relevance in medical education; (3) functional diversity covering text, image, video, and domain-specific tasks; and (4) market prevalence, wide recognition, technical maturity, and development by prominent AI companies. Notably, models like Perplexity, DeepSeek, Notebook LM, and Midjourney, though used by clinicians and students in specific scenarios, were not included due to limited evaluative data and insufficient supporting information in the referenced reports.

**Figure 1.**
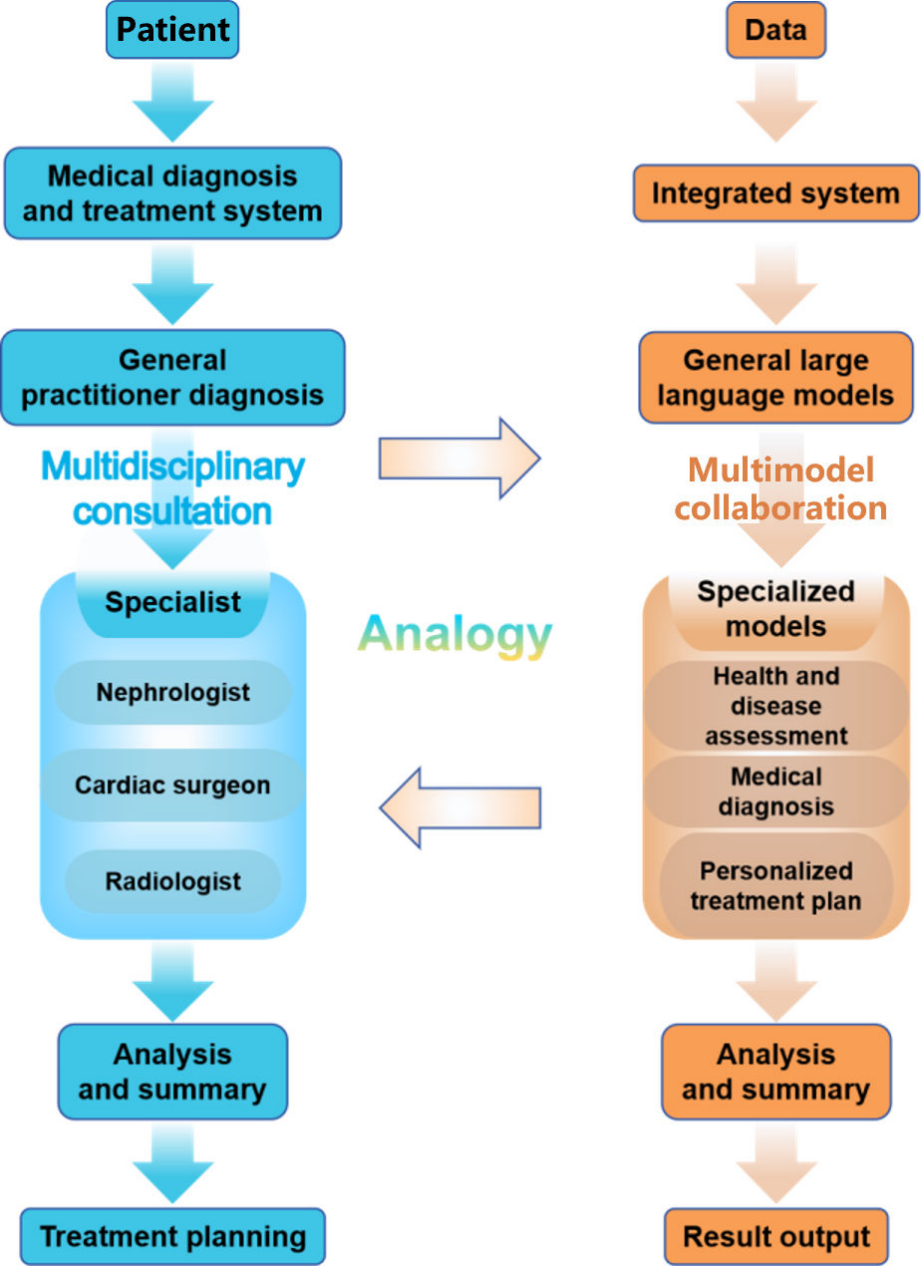
Specialized model integration system based on general large language models.

Within this ecosystem, general LLMs serve as multitasking hubs, leveraging cross-domain adaptability and natural language interaction, while specialized models achieve context-specific efficacy through the embedding of deep medical knowledge. To resolve their complementary yet fragmented coexistence, we propose a specialized model integration system anchored to general LLMs, inspired by symbiotic agency theory and hospital diagnostic workflows [21] (see Figure 1). This architecture establishes a 3-tiered clinical analog: general LLMs serve as primary coordinators, managing task orchestration; specialized models act as domain experts, executing depth-specific processing; and protocol-based collaboration enables online consultation through knowledge distillation and output validation. This hierarchical integration embodies symbiotic agency principles: general models extend the applicability of specialized techniques by transcending domain boundaries, while specialized models enhance system depth by reinforcing medical logical rigor. Through functional complementarity and role differentiation, they form a synergistic symbiont exceeding individual capability limits, establishing an intelligent foundation for medical education characterized by adaptability, expertise, and reliability.

#### Conceptual Model 2: Tripartite Synergistic Integration Model for Medical Education Resources, Methods, and Assessment

**Figure 2.**
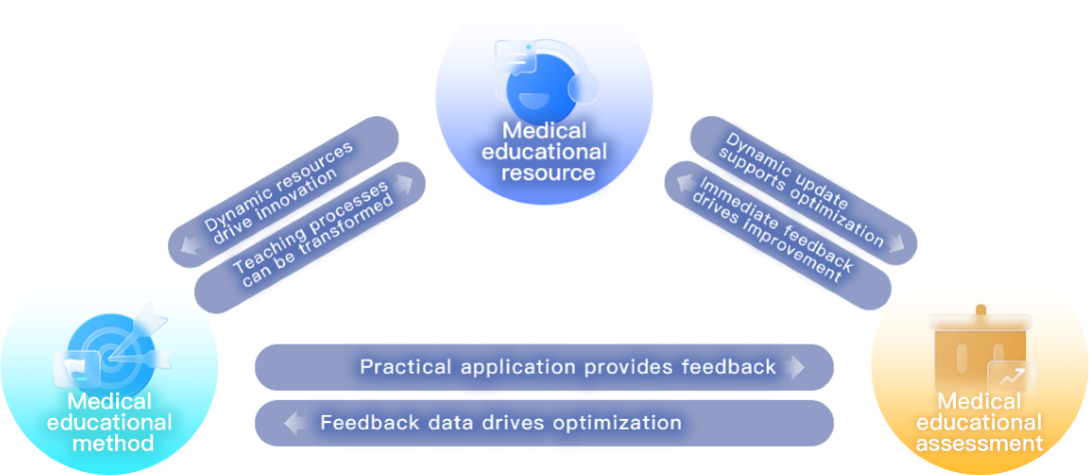
The model of integrated and collaborative development of medical education methods, resources, and assessment.

The tripartite synergy paradigm, rooted in complex systems management theory and evidenced across domains from political governance to integrated health care systems (eg, the mission alignment model by Peek et al [22]), establishes our resource-method-assessment (RMA) framework (see Figure 2) as the core analytical structure [22,23]. This framework defines three interdependent dimensions: (1) resources encompassing dynamic content provisioning mechanisms, (2) methods designing knowledge-to-practice training pathways, and (3) assessment managing outcome monitoring and feedback generation. Their cyclical optimization forms an integrated whole, as resource renewal enables pedagogical innovation, method implementation yields evaluative data, and assessment outputs drive resource refinement and method calibration. Within this architecture, GAI operates as a collaborative instrument executing content generation, interaction support, and data analysis under educator-directed goal design, ethical governance, and critical intervention. The established framework provides essential categorization criteria for subsequent empirical analysis: it defines 3 dimensions—resources, methods, and assessment—directly corresponding to 3 primary research domains in GAI applications for medical education. By consolidating fragmented literature within a unified analytical structure, this framework systematically addresses cognitive limitations arising from isolated examinations of technological functions, thereby elucidating the intrinsic operational logic of technology-enabled educational transformation.

## Methods

### Review

With the rapid development of GAI, its applications in medical education have garnered considerable attention and have become a significant research focus. We conducted a preliminary search using the keyword combination of “generative artificial intelligence” and “medical education” across PubMed, Web of Science, and Scopus. Our goal was to analyze the publication trend regarding the applications and challenges of GAI in medical education over the past 5 years (from January 2020 to October 2024). The literature search was limited to sources published between January 2023 and October 2024 for the following reasons: (1) Technological progression: The 2023‐2024 period coincides with a shift from theoretical proposals (pre-2023) to empirical studies on GAI implementation in medical education. (2) Scope alignment: The review prioritizes analysis of current applications, identified limitations (eg, output inaccuracies and integrity concerns), and near-future developments rather than historical trends. (3) Avoiding redundancy: Pre-2023 literature is excluded to prevent overlap with existing syntheses and focus on emergent applications (eg, automated assessment and adaptive resources) evidenced in the sampled literature (n=131). (4) Practical relevance: This timeframe reflects consolidated evidence on operational challenges and benefits relevant to contemporary pedagogical decision-making.

### Search Strategy

We used Boolean operators to combine GAI and medical education keywords, creating the final search strategy (see Multimedia Appendix 2). A thorough search was conducted across 3 major databases: PubMed, Web of Science, and Scopus, focusing only on English-language articles published from January 2023 to October 2024.

### Inclusion and Exclusion Criteria

This study included research articles focusing on the applications, challenges, and future development of GAI in medical education applications. Articles were excluded if they were commentaries, editorials, viewpoint, perspective, and short reports or communications with low level of evidence or did not discuss GAI within medical education. Studies focusing on non-GAI forms such as predictive analytics and natural language processing or those centered on other fields of medical education (eg, nursing) were also excluded. We excluded nursing based on fundamental educational differences. Clinical and dental education follow structured undergraduate curricula focused on acute care, diagnostics, and technical skills within hospital settings. Nursing emphasizes community practice, longitudinal relationships, and chronic disease management [24]. Including nursing would introduce significant heterogeneity in learning outcomes, GAI applications, and educational contexts. This methodological exclusion preserves thematic coherence and internal validity for analyzing GAI’s role in comparable, technology-driven medical education environments.

Initially, YL and ZL conducted a preliminary screening of titles and abstracts from 3 databases. With the help of Zotero 7.0.13 (64-bit), a document management software (it is a project of Digital Scholar and developed by a global community), ZL detected duplicates of the initially screened articles according to title, author, abstract, and other information and removed duplicates. Following this initial phase, YL and ZL independently reviewed the full texts for a second round of evaluation. In cases of disagreement, ZY and NZ were consulted to mediate and make the final determination regarding inclusion.

### Data Extraction Protocol

To ensure the systematicity, transparency, and reproducibility of this scoping review, a detailed data extraction protocol was developed and rigorously followed.

#### Data Point Definition and Protocol Development

Before comprehensive data extraction, a structured data extraction form was collaboratively developed by all authors. This iterative process was guided by our research questions and the predefined thematic framework outlined in Table 1, which focused on the applications, challenges, and prospects of GAI in medical education applications. The form was designed to systematically capture key information from each included article, encompassing: bibliographic details (eg, authors, publication year, journal, and country or region), study characteristics (eg, research design, objectives, and population), specific GAI models used (eg, ChatGPT [OpenAI] and Gemini [Google]), application scope (single-model vs multimodel), analysis type (performance comparison across models or examination of synergistic enhancement through model integration)**,** detailed descriptions of identified applications, challenges, and future directions of GAI application in medical education categorized exclusively through our tripartite Trinity Framework and quantitative performance metrics (reported accuracy rates, percentages, mean scores, standard deviations, and *P* values related to GAI model performance in various tasks). This granular definition of data points ensured that all relevant information pertinent to our broad research inquiry was systematically collected.

**Table 1.** A systematic thematic analysis of applications and challenges of generative artificial intelligence (GAI) in medical education.

Category and theme		Subtheme
Medical educational assessment		
	Scoring short answers automatically.	—^a^
	Evaluating articles.	—
Medical educational resources		
	Providing standard answers.	The performance of different question types. The performance of different difficulty questions. The performance of questions at different cognitive levels.
	Generating diverse clinical cases.	—
	Digital interaction and communication training.	—
	Sharing educational resources.	—
	Generating clinical images.	—
Medical educational methods		
	Curriculum design.	—
	Generating customized teaching aids.	—
	Generating explanations for MCQ^b^.	—
	Personalized learning support.	—
	Medical decision aid.	—
	Multidisciplinary knowledge acquisition.	—
	Academic writing optimization.	—
Existing defects at this stage		
	Insufficient scene adaptability.	Poor ability to handle complex clinical scenarios. Lack of local background in specific regions. Language adaptability issues. Lack of nontextual information analysis skills.
	Data quality and information bias	Hallucination phenomena. Lack of details on output content. Lack of personalization. Dataset dependency.
Potential issues in the future		
	Overreliance	Impaired critical thinking. Decreased creativity. Decreased teamwork ability. Decreased practical problem-solving ability.
	Ethical controversy	Authenticity of the test results. Academic misconduct. Lack of clinical interaction and emotional resonance. Resource inequality. Ownership of intellectual property rights. “Black box” problem and attribution of responsibility.

a Not available.

b MCQ: multiple choice question.

To better understand the global research landscape in this field, we analyzed the countries or regions of origin for the 131 selected articles. For those without a precise location, we assigned them according to the country or region of the corresponding author’s institution. To analyze the distribution of research based on the countries or region’s development level, we used the Human Development Index (HDI) classification. The latest HDI data categorizes countries or regions into 4 tiers: very high, high, medium, and low human development with higher HDI scores correlating with greater national development. We also investigated cross-level HDI collaborations, which refer to partnerships between countries from different HDI categories [25].

#### Protocol Testing and Quality Control

To validate the comprehensiveness and clarity of the data extraction form, a pilot test was independently conducted by 2 reviewers, YL and ZL, on a randomly selected subset of 10 included articles. During this pilot phase, any discrepancies in data extraction or ambiguities within the form were identified and discussed. Based on these discussions, the data extraction form underwent iterative revisions to refine categories, clarify definitions, and ensure consistent interpretation of data points among reviewers. Following this refinement, YL and ZL independently extracted data from all 131 included articles. In cases of disagreement between the 2 independent extractors, consensus was initially sought through discussion. If a consensus could not be reached, a third and fourth reviewer, ZY and NZ, were consulted to mediate and make final determinations regarding the applicability and extraction of the data.

### Synthesis of Results

ZY subsequently compiled and reorganized the extracted data, assigning new identifiers for easier reference. This organized dataset was then categorized according to the predefined themes and subthemes (see Table 1), forming the basis for the subsequent descriptive summary and analysis. Our analysis employed a theory-driven, top-down approach anchored in a tripartite conceptual model of medical education: resource generation, method innovation, and assessment upgrade. The following sections will present a descriptive summary of the extracted data.

## Results

### Overview

**Figure 3.**
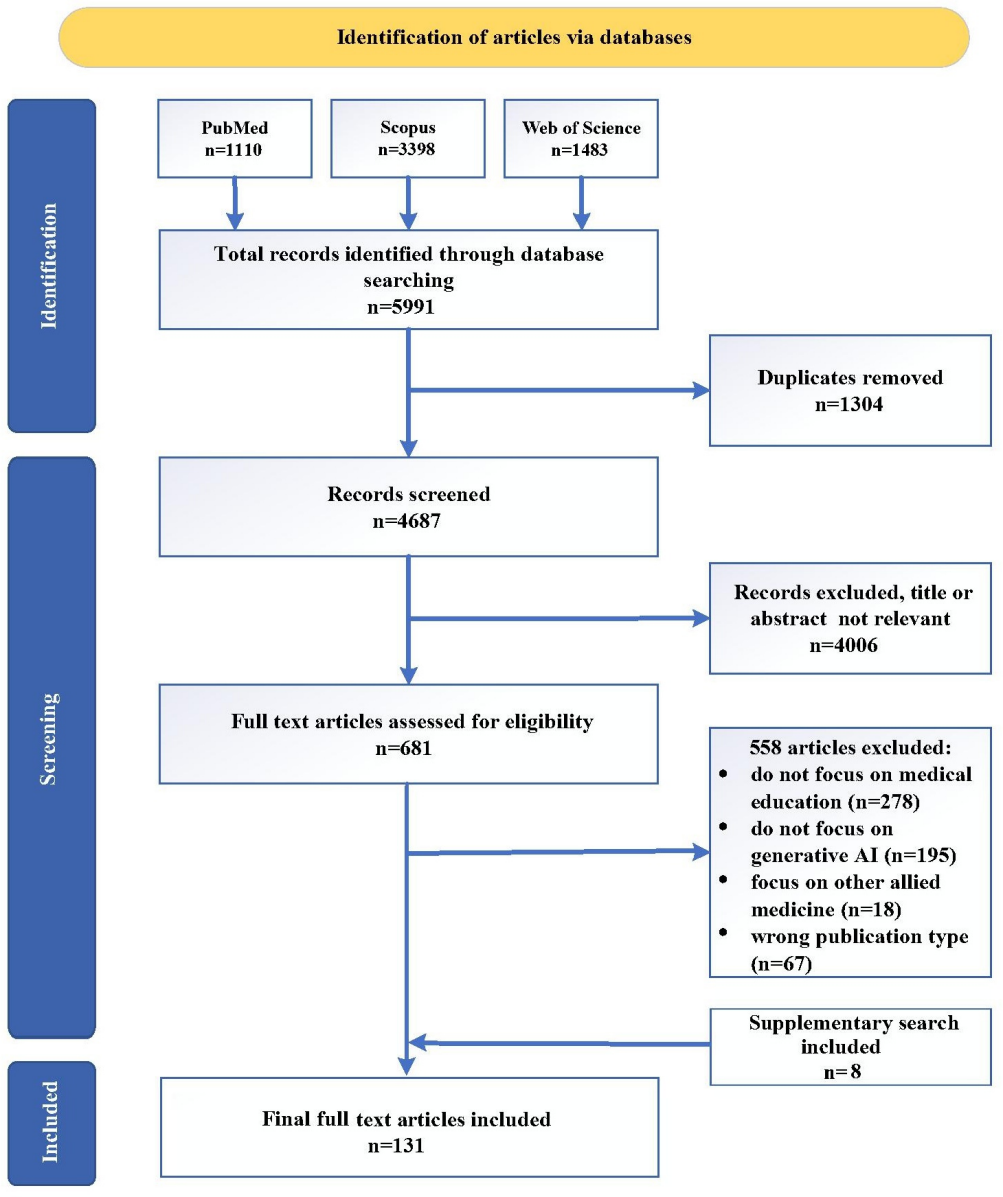
Article screening flow chart. AI: artificial intelligence.

Following our search strategy, we retrieved 5991 articles, of which 1304 were duplicates, leaving 4687 articles. In the first round of screening, 4006 irrelevant articles were excluded based on titles and abstracts, leaving 681 articles. In the second round, we excluded 558 articles after full-text review, including 278 nonmedical education articles, 195 non-GAI articles, 18 focused on other medical fields (eg, nursing), and 67 of different types (eg, commentaries). During the paper preparation, we conducted a supplementary search for 8 systematic reviews and meta-analyses. Ultimately, 131 articles were included in the final review (see Figure 3). Among the 131 included studies, the distribution of research designs was as follows: 83 cross-sectional studies, 5 randomized controlled trials (RCTs), 2 quasi-experimental studies, 1 cohort study, 1 quasi-randomized controlled trial, 8 systematic reviews and meta-analyses, 5 mixed-methods studies, and 1 case study. The remaining 25 studies were categorized as “other” with nonstandardized research designs, which were not fitting typical epidemiological or evidence-based medicine classifications. Collectively, cross-sectional studies (descriptive research designs) constituted the majority (n=83), reflecting the emerging state of GAI in medical education, where most research focuses on initial application explorations, feasibility assessments, and user experience descriptions rather than hypothesis-driven experimental designs. Other study types, including RCTs, cohort studies, and systematic reviews, provided supplementary evidence on intervention effects, longitudinal trends, and synthesized findings, respectively.

### Analysis of Literature Source and Human Development Levels

**Figure 4.**
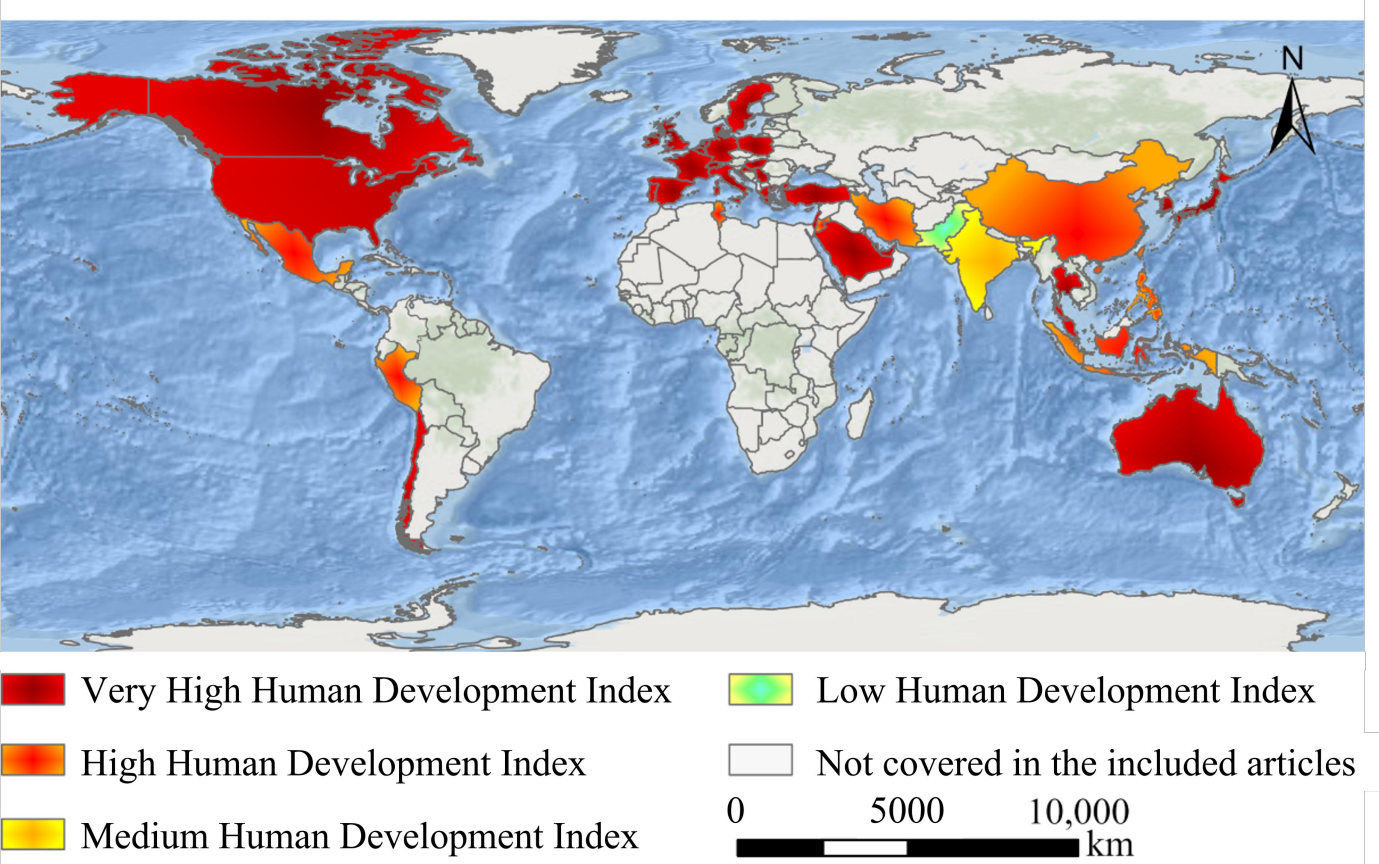
Geographical distribution of countries or regions of origin for generative artificial intelligence (GAI) research in medical education.

Based on the countries or regions of origin for the included articles and HDI classification, we analyzed the distribution of related studies. The results are illustrated in Table 2 and Figure 4. A significant portion (74%, n=97 articles) of the research came from countries or regions with very high human development, with the United States contributing 33 studies. High human development countries or regions produced 15% (n=19 articles), with China contributing 13 studies. Medium human development countries or regions contributed 5% (n=7 articles), mainly from India, while low human development countries or regions accounted for only 2% (n=3 articles). Furthermore, 4% of the studies (n=5 articles) involved cross-level collaborations, primarily between very high and medium or low HDI countries or regions.

**Table 2.** Distribution of countries or regions of origin for generative artificial intelligence (GAI) research in medical education (categorized by the HDI^a^).

HDI classification	Portion, n (%)
Very high human development	97 (74.0)
High human development	19 (14.5)
Medium human development	7 (5.3)
Low human development	3 (2.2)
Cross-level HDI collaboration	5 (3.8)

a HDI: Human Development Index.

### Applications of GAI in Medical Education

#### Medical Educational Assessment

##### Scoring Short Answers Automatically

A recent study examined GPT-4 (OpenAI) and Gemini 1.0 Pro in automated short answer grading using 2288 student responses from 12 undergraduate medical courses across 3 languages, with instructor-provided rubrics or sample solutions as reference standards. GPT-4 showed high precision (0.91) in identifying fully correct answers, though its scores were significantly lower than human graders, while Gemini 1.0 Pro had no significant difference from human evaluations, with a mean normalized score of 0.68 (SD 0.32) and median of 0.75, similar to humans. Both models demonstrated high consistency across repeated evaluations, especially with high-quality standard responses, and these findings are specific to undergraduate medical education contexts [26].

##### Evaluating Articles

Liu et al [27] reported that in their study of 50 rehabilitation-related original articles, 50 sections (introductions, discussions, and conclusions) were generated by ChatGPT-3.5 and 50 were corresponding AI-rephrased versions using Wordtune Originality.ai, achieved 100% accuracy in detecting both AI-generated and AI-rephrased content. ZeroGPT correctly identified 96% of AI-generated texts and 88% of rephrased ones. The study focused specifically on rehabilitation medicine with analyzed content limited to partial article sections rather than full texts. It is notable that such high detection rates have not been widely observed across other disciplines or with newer large language model versions. The specialized nature of medical writing, including technical terminology use, may also influence these outcomes in ways not seen in broader academic contexts, which should be considered when evaluating the generalizability of these findings. Another study comparing automatic scoring systems (ChatGPT-3.5 and ChatGPT-4) with manual scoring for article quality assessment found no significant difference between GPT-4-based scoring and human grading. This demonstrates the considerable potential of GAI to enhance the quality evaluation of articles [28].

### Medical Educational Resources

#### Providing Standard Answers

The performance of different question types: The studies encompassed a range of question types, including multiple-choice questions (MCQs), single-choice questions, short-answer questions (SAQs), true or false questions, open-ended short-answer questions (SOAQs), long-answer questions, clinical case analysis questions (CAQs), and image-text integrated questions [29-37]. An exploratory study conducted by a research team from Qatar University evaluated ChatGPT’s performance across various assessment formats relevant to undergraduate dental education. The study included 50 assessment items covering 50 different learning outcomes, with 10 items for each of the 5 formats: MCQs, SAQs, short essay questions (SEQs), single true or false questions, and fill-in-the-blank items. These items were based on core clinical topics in dental education, such as restorative dentistry, periodontics, endodontics, and oral surgery, aligned with the learning outcomes expected of undergraduate dental students. In this study, ChatGPT demonstrated 90% accuracy for SAQs, SEQs, and fill-in-the-blank items and notably achieved 100% accuracy in single true or false questions [31]. However, other studies have revealed a significant decline in accuracy for CAQs, as low as 17%, which require strong logical reasoning and lack predefined options [34].

Regarding MCQs, a study reported that GPT-4 and Microsoft Bing achieved top scores (76%) on the University of Antwerp medical licensing MCQ exam, outperforming medical students. However, ChatGPT’s accuracy fell considerably when tackling Chinese-language medical MCQs with an accuracy of 37%. In addition, another study reported that in the Chinese Master’s Degree Entrance Examination, ChatGPT’s accuracy for single-choice questions (A1 type) was 56%, whereas for MCQs, it dropped to 33% [15]. These findings suggest that GAI’s performance is not uniformly robust across all MCQ types and is influenced by factors such as question structure, subject domain, difficulty, language, and the presence of clinical vignettes or images.

The performance of different difficulty questions: In terms of difficulty, questions were generally classified as “easy,” “medium,” or “difficult.” For example, the difficulty levels are defined based on the performance indicators of the historical question bank: “Difficult” (*P*<.30; less than 30% of the students answered correctly), “Medium” (*P*=.30 to .80), and “Easy” (*P*>.80) [35]. ChatGPT-4 demonstrated strong performance on easy questions, with accuracy rates reaching 97.4%. Yet, even in this category, ChatGPT-4’s performance lagged behind that of residents [35,38-42]. In contrast, ChatGPT-4 excelled on medium and difficult questions, outperforming residents by 25.4 and 24.4 percentage points, respectively [38]. Across all models, performance tended to decline with increased difficulty, especially for higher-level questions that required multistep reasoning, where accuracy dropped markedly [39-45].

The performance of questions at different cognitive levels: 2 studies investigated the performance of ChatGPT-4 on questions categorized by Bloom’s taxonomy, which includes 6 cognitive levels: remembering, understanding, applying, analyzing, evaluating, and creating [46]. These studies found that ChatGPT-4 consistently performed well across all cognitive levels, with an average correct answer rate of 71.96% for each cognitive level [47,48].

#### Generating Diverse Clinical Cases

By collaborating with instructors, GAI can quickly generate comprehensive clinical cases, including patient history, physical examination results, lab data, and differential diagnoses tailored to predefined learning objectives (eg, chest pain and joint pain). This reduces the time instructors spend developing such cases [49,50]. Furthermore, GAI-generated cases can integrate various contextual factors such as race, occupation, and lifestyle, significantly enriching the diversity of teaching materials [51]. For example, when creating a case based on a disease profile specific to a region, the ethnicity of the generated patient can be adjusted accordingly. In the context of type 2 diabetes, modifications can be made to the age range and weight distribution. In addition, randomized prompts for urine analysis may be included in urinary tract infection cases. Both patient presentations and examination findings can be randomized, and symptom expression can be customized to meet specific learning needs [51].

In Smith and colleagues’ [52] study, GAI was assigned the task of creating a case of an immigrant with mental health concerns, as this group may require specialized social psychiatry interventions. The results indicated that GAI was able to produce a case that met fundamental educational objectives. However, it included several signs of emotional disorders, highlighting a need for further refinement.

#### Digital Interaction and Communication Training

Studies have shown that GAI is effective in promoting interactive learning and providing practice in communication skills. GAI-powered simulation tools simulate changes in clinical conditions in scenarios such as advanced cardiac life support (ACLS) and intensive care unit (ICU) sepsis, prompting students to critically analyze whether their decisions are correct [53]. In addition, conversational GAI-created digital patients provide anesthetists with valuable training for patient interactions, reducing reliance on human actors while enhancing the flexibility and consistency of the training process [54]. These digital interactions create a safe space for repeated practice, providing dynamic learning experiences that traditional textbooks cannot match [52,55]. Furthermore, conversational GAI models, such as chatbots, can simulate the role of a professor, offering critical evaluations of literature and distilling complex research into easily understandable key findings, thus fostering simulated discussions between students and experts in the field [56]. However, besides experiences and qualitative observations, formal evaluations of the reliability and validity of such GAI-generated information in a professor-like capacity are still needed.

#### Sharing Educational Resources

By generating accessible public health information, GAI enhances the public’s understanding of essential health issues, such as infectious disease prevention and vaccination, ultimately leading to improved health literacy [30]. Furthermore, GAI-generated clinical cases can be disseminated as Open Educational Resources (OERs), providing medical educators with globally adaptable teaching materials that are customized to local contexts [51].

#### Generating Clinical Images

GAI tools such as Adobe Firefly, DALL·E 2 (OpenAI), Bing Image Creator, and generative adversarial networks (GANs) can create clinical images displaying various pathological features based on textual descriptions, potentially addressing the shortage of authentic pathological images in traditional medical education due to medical confidentiality and patient privacy restrictions [51,57-59]. For instance, images of retinal disease generated by the stable diffusion model enhance students’ learning opportunities in ophthalmic pathology, greatly enhancing the availability of visual teaching resources [57]. However, their reliability and accuracy vary significantly across models and tasks. For example, DALL·E 2 demonstrated an overall clinical accuracy rate of 22.2% in aligning generated images with textual prompts across 15 semantic relations (eg, spatial and action-based relationships), with only 3 relationships (touching, helping, and kicking) achieving moderate consistency above 25%. In a medical education context, DALL·E 2 achieved 78% accuracy for soft-tissue tumor images but produced inconsistent results for wound images, with 65% of generated wound images containing anatomical inaccuracies or irrelevant elements [58]. A comparative study of DALL·E 2, Midjourney, and Blue Willow for generating skin ulcer images showed DALL·E 2 performed best with an average score of 3.2/5 (scale 1‐5) but still produced irrelevant content (eg, X-rays instead of pressure ulcers) in 20% of cases. Midjourney generated stylized, exaggerated features in 40% of images, while Blue Willow produced images with little relevance to prompts in 70% of attempts [59].

### Medical Educational Methods

#### Curriculum Design

GAI shows potential in the early stages of curriculum development, aiding in quickly creating course objectives, learning strategies, and frameworks. For instance, in a study on integrated pharmacotherapy of infectious disease education modules, ChatGPT helped design curriculum goals (eg, “describe mechanisms of antibiotic resistance”) with an average expert rating of 92% for appropriateness and accuracy, supporting educators—especially in designing foundational courses [60].

#### Generating Customized Teaching Aids

Researchers have developed derivative applications based on classical models, such as Glass AI (a powerful AI-driven knowledge management system developed by Glass Health, focusing on organizing and retrieving health-related information efficiently). It integrates GPT-4 with evidence-based, peer-reviewed clinical guidelines to generate differential diagnoses and clinical plans based on textual input of clinical cases, enabling students to interact with it and experience the GAI-driven diagnostic process for cases [61]. Similarly, an MCQ generator based on ChatGPT-generated cases offers a dynamic platform for personalized learning assessments [62].

#### Generating Explanations for Multiple-Choice Questions

Research shows that when GAI is used to answer MCQs, the explanations generated by GAI can better convey key knowledge points and achieve good accuracy and degree of matching with teachers’ explanations. Of the 81 questions explained by the teacher and correctly answered by ChatGPT, 92.6% of the explanations were accurate and included at least part of the teacher’s explanation. However, the research also highlights that if an initial response is incorrect, the likelihood of subsequent errors increases significantly (*P*<.001), indicating that an early mistake may lead to systematic inaccuracies in later explanations [63]. Complementing this, in a systematic review, the broader literature reviewed showed that the majority of studies (5/8, 62.5%) indicate the effectiveness of AI in generating valid MCQs, with a preference for the latest GPT-4 models (6/8, 75%) [64].

#### Personalized Learning Support

Studies demonstrate that GAI boosts students’ learning efficiency across multiple stages by offering personalized feedback and customized content. This includes support for exam preparation [55,65-68], optimizing learning paths and review strategies [52,69-71], clarifying medical concepts [68,72-76], and assisting in the development of tailored career plans [77]. For instance, in physiological case analysis, GAI offers precise responses and contextually relevant feedback. A cross-sectional study tested 77 physiology case vignettes (covering diverse physiological and pathophysiological scenarios, designed for undergraduates) on ChatGPT 3.5, Google Bard, and Microsoft Bing. Rated by two physiologists on a 0‐4 scale, ChatGPT scored highest at 3.19 (SD 0.3), outperforming Bard (2.91, SD 0.5) and Bing (2.15, SD 0.6) with *P*<.001. ChatGPT’s precision accelerates task completion, helping students grasp medical knowledge in practical scenarios more effectively [78]. Furthermore, a study found that in cases of initial incorrect responses, GPT-4 was able to self-correct and provide accurate answers after simple follow-up questions or hints, mimicking pedagogical interactions observed in residency programs. This dynamic learning approach, coupled with rapid information processing, positions GPT-4 as an important asset for personalized learning [79].

#### Medical Decision Aid

GAI uses its ability to analyze complex, domain-specific knowledge to support the diagnosis of rare and intricate diseases. In addition to diagnosis, it can generate differential diagnoses tailored to the unique characteristics of each disease, providing health care professionals with precise decision-making support [80-83]. For common pathological issues and basic data analysis, GAI tools are efficient and accurate, helping pathologists organize their thought processes and expedite the initial diagnostic phases [84]. The impact of domain-specific training is profound. For instance, refined datasets in the surgical and anesthesiology fields enhance GAI’s clinical decision-making capabilities. In scenarios such as a “30-year-old pregnant woman requiring an emergency appendectomy,” GAI suggests not only tailored surgical strategies but also factors in critical anesthesia protocols [85]. Furthermore, in the field of traditional Chinese medicine, when combined with such tools, GAI can effectively create knowledge maps that organize entities, attributes, and their relationships to traditional Chinese medicine through graphical structures. GAI provides unique support for teaching traditional Chinese medicine and disease diagnosis and treatment decisions [86].

#### Multidisciplinary Knowledge Acquisition

GAI demonstrates potential in multidisciplinary knowledge acquisition within medical education by providing high-quality knowledge across various medical subfields [87-94]. GAI demonstrates adaptability across disciplines, including shoulder and elbow surgery, sports medicine, and oncology [91]. Research further indicates that GAI models such as ChatGPT-4 excel in internal medicine, pediatrics, obstetrics and gynecology, surgery, emergency care, and public health [88-90,92-94]. Notably, a study assessing ChatGPT-4’s performance in the American Board of Family Medicine (ABFM) certification examination demonstrated its significant proficiency, with both the custom robot version (embedded in a specialized subenvironment designed to mimic examination conditions and given extensive preparation resources) and the regular version (standard ChatGPT-4) achieving high correct response rates of 88.67% and 87.33% respectively, well above the passing threshold. This further highlights GAI’s value in enhancing medical education within a multidisciplinary framework, making it a powerful learning support tool across a wide range of fields, including family medicine [95]. A meta-analysis of ChatGPT-3.5/4 across medical, pharmacy, dentistry, and nursing licensing exams revealed an overall accuracy of 70.1% (95% CI 65%‐74.8%; *P*<.001). Performance varied significantly by field (Q=15.334; *P*=.002), with pharmacy having the highest rate (71.5%, 95% CI 66.3%‐76.2%) and nursing having the lowest rate (61.8%, 95% CI 58.7%‐64.9%). These results demonstrate GAI’s potential to provide multidisciplinary learning support in health professions [96]. It is crucial to note that the evidence presented in this section highlights the individual learner’s ability to access and comprehend information across disciplines. This review’s existing evidence has not yet extensively covered GAI’s direct support for complex interdisciplinary teamwork, closed-loop communication, or the cultivation of specific professional behaviors within collaborative learning environments.

#### Academic Writing Optimization

A study shows that GAI excels in creating article outlines and editing formatting, which alleviates common writing challenges related to poor organization and grammatical mistakes [28]. In addition, GAI can significantly improve the quality and standardization of academic writing, allowing medical educators and students to express their ideas more accurately and clearly [28,55,97]. Furthermore, GAI assists students in organizing and generating literature content while writing their thesis [98]. The content produced by GAI maintains consistency in language and includes appropriate academic terminology and logical structure, helping students present themselves more professionally in their academic writing [55]. Furthermore, GAI supports many non-native English speakers in overcoming language barriers during the academic writing process, which enables them to engage more confidently in academic communication [71].

### Statistical Analysis of the Application of GAI Models

The models discussed in 131 articles include ChatGPT, Gemini (formerly known as Bard), Copilot (formerly known as Bing), Claude, and LLaMA, as well as other types of models such as StyleGAN2-ADA, Stable Diffusion, and customized chatbots.

Among the various models studied, ChatGPT stands out due to its advanced natural language processing capabilities. Of the 131 articles, 119 (89.5%) focused on ChatGPT, which was applied in diverse educational contexts, including simulating doctor-patient conversations, generating exam questions, and providing personalized learning support. These applications highlight their flexibility and adaptability in medical education. Notably, research had shown that as versions have iterated, ChatGPT-4 has significantly improved in both performance and scope compared to ChatGPT-3.5 [26,94,99-101].

Gemini was mentioned in 22 articles, accounting for 16.5% of the total. Copilot was mentioned in 11 articles, primarily due to its integration with the Microsoft ecosystem, making it ideal for educational management and resource development. Claude was cited in 6 articles. LLaMA, referenced in 4 articles, stands out for its ability to run locally, making it suitable for environments with limited resources. In addition, StyleGAN2-ADA, Stable Diffusion, and Convai were discussed in individual studies, mainly for their use in image generation and visualizing doctor-patient interactions.

The performance assessment of two or more models was compared in 26 articles. In comparative studies within the articles, numerous models have undergone head-to-head research, including ChatGPT-4 with Gemini 1.0 Pro [26], ChatGPT-4 with ChatGPT-3.5 [96], ChatGPT 3.5 with Google Bard and Microsoft Bing [78], DALL-E 2 with Midjourney and Blue Willow [59], and Originality.ai with ZeroGPT [27]. Based on these head-to-head investigations, different models demonstrate proficiency in specific tasks: ChatGPT-4 performs better in handling complex tasks, providing accurate medical knowledge, generating exam questions, and offering personalized learning support, especially in English-language medical licensing examinations; Gemini 1.0 Pro is noted for its strong contextual understanding and multimodal capabilities; ChatGPT-3.5 excels in simulating doctor-patient conversations, generating exam questions, and providing personalized learning support; Microsoft Bing achieved top scores alongside GPT-4 in medical licensing MCQ exams; DALL-E 2 shows potential in creating clinical images with specific pathological features from textual descriptions; and Originality.ai achieves high accuracy in detecting both AI-generated and AI-rephrased medical writing.

### Challenges of GAI in Medical Education

#### Existing Defects at This Stage

##### Insufficient Scene Adaptability

Insufficient scene adaptability is due to the following factors.

First is the poor ability to handle complex clinical scenarios. GAI faces substantial limitations when handling complex clinical scenarios, particularly in cases requiring multistep reasoning, intricate calculations, and recognition of atypical clinical symptoms [45,87,102,103]. For instance, studies have shown that GAI struggles with MCQs, X-type problems, and tasks demanding deep reasoning. This underscores its limited ability to perform the nuanced decision-making required in medical judgments [29,30,39,41,44,47,48,66,67,84,89-92,104-107]. Furthermore, GAI-generated clinical scenarios often lack flexibility and fail to replicate the diversity and complexity of real-life clinical environments, thereby limiting learners’ exposure to the spectrum of challenging cases [108,109]. GAI also faces technical limitations in generating simulated images for complex diseases, resulting in images that fail to depict atypical manifestations accurately [57]. Furthermore, GAI models demonstrate uneven knowledge depth, exemplified by an ophthalmology meta-analysis: accuracy was 78% in “Pathology” but significantly lower in foundational or clinical areas, such as “Ophthalmology fundamentals” (52%), “Clinical ophthalmology” (57%), and “Refractive surgery” (59%) [110].

Second is the lack of local background in specific regions. Numerous studies have shown that GAI often struggles to adapt effectively to a specific region’s unique background and needs when dealing with medical content related to that region, thereby undermining its universal applicability in multicultural settings [33,38,102,111]. For example, ChatGPT often responds to public health issues in India with a Western-centric perspective, overlooking local situations and cultural differences [33]. Similarly, ChatGPT struggles to accurately comprehend and adapt to the local regulatory environment when addressing medical policies specific to China, largely due to the limited representation of Chinese data in its training set [102,112].

Third is language adaptability issues. Currently, GAI exhibits significant limitations in processing languages, particularly in non-English medical education environments. The accuracy of GAI models like ChatGPT often varies greatly when handling languages such as Chinese, Korean, and Polish, resulting in incorrect outcomes in these contexts [29,30,34,38,106,113-115]. A meta-analysis quantified disparity: GPT-3.5 achieved 57% accuracy (95% CI 52%‐62%; *P*<.01) in English-speaking countries and 58% (95% CI 52%‐64%; *P*<.01) in non-English-speaking countries (*P*=.72). GPT-4 scored 86% (95% CI 82%‐89%; *P*<0.01) in English-speaking countries versus 80% (95% CI 76%‐83%; *P*<.01) in non-English-speaking countries (*P*=.02), demonstrating the adaptability issues of GAI models across different linguistic and regional contexts [116].

Fourth is a lack of nontextual information analysis skills. Current GAI tools like ChatGPT and Bard struggle to handle image-based queries, limiting their application in fields such as dentistry, neurosurgery, and nuclear medicine, where visual analysis of images and tissue samples is crucial for clinical decision-making [31,36,42,67,73-75,117].

##### Data Quality and Information Bias

Data quality issues and information bias occur due to the following factors.

First is the hallucination phenomenon. In GAI applications, hallucinations occur when the content generated by GAI diverges from factual accuracy or contradicts itself, remaining a prevalent issue. In total, 3 primary types of hallucinations have been identified: input-conflicting hallucination, context-conflicting hallucination, and fact-conflicting hallucination. Input-conflicting hallucination occurs when the GAI-generated content contradicts the initial information provided by the user. This can mislead learners and hinder their understanding of specific concepts [51,65,118]. Context-conflicting hallucination arises when the GAI offers contradictory responses to the same or similar questions. This inconsistency is particularly evident in complex case analyses [71,90,119,120]. Fact-conflicting hallucination occurs when the GAI reports facts that contradict established information, often with a high confidence level, which can easily mislead learners [54,121-137].

Second is the lack of details on output content. Numerous studies have highlighted that GAI often generates overly simplified or vague responses, lacking essential details and knowledge necessary for a comprehensive understanding [31,53,56,60,63,73,114,118,120,135,136,138,139]. For instance, evaluations of GAI in cardiology have revealed that it fails to specify the types of heart murmurs associated with valve diseases. In addition, GAI-generated descriptions of pathophysiology and epidemiology tend to be overly general, often including vague statements such as “certain age groups are at higher risk” without specifying the specific conditions. Furthermore, GAI often produces incomplete or inaccurate information when generating case study materials, which can lead to misleading students. For example, GAI-generated learning materials on melanoma have been known to omit crucial tumor markers like S-100 or the latest treatment for BRAF (B-Raf proto-oncogene, serine, or threonine kinase) mutations [63,138]. The same problems are evident in academic writing assistance, where GAI may create basic article structures but often lacks the depth, detail, and critical citations found in human-generated content [120].

Third is the lack of personalization. The content generated by GAI lacks personalization tailored to individual needs. This limitation mainly manifests in the generated text, which often adopts similar writing patterns and standardized language, struggling to incorporate personalized perspectives or creative expressions [28]. In a medical environment, GAI-generated treatment plans, although generally reasonable, often fail to consider individual patient characteristics, such as the severity of the disease, lifestyle, and personal preferences [105].

Fourth is dataset dependency. The performance of GAI is significantly influenced by the quality and diversity of its training data. If the data is insufficient or skewed, it may lead to potential biases and limitations in practical applications, causing underperformance in less-represented areas [33,59,73,82,85,86,111,117,122,126,140-143]. In addition, the cutoff date for the training data means that GAI may lack knowledge of the latest research, leading to outdated or inaccurate recommendations [26,32,41,66,67,74,80,89,92,94,109,129,136,141,144]. For example, when advising on treatment for bipolar disorder in pregnant women, ChatGPT-4 failed to incorporate the latest studies and instead suggested outdated methods [89]. Furthermore, the data bias present in GAI during the training process cannot be overlooked [51-53,58,61,71,72,78,107,108,132,139,145]. Such biases often arise from the intrinsic imbalances within the dataset, which subsequently permeate the generated content. These biases manifest as stereotypes, mainly depicting certain professions or physical attributes. For instance, some occupations may be associated with higher BMIs, while the French ethnicity is stereotypically linked to the profession of “wine connoisseur” [51].

### Potential Issues in the Future

#### Overreliance

Overreliance can be caused due to the following factors.

First is impaired critical thinking. The rapid feedback provided by GAI may reduce students’ time for deep thinking, weakening their ability to analyze problems and independently engage in critical learning. This phenomenon is particularly evident in medical education, where students often rely on the answers provided by GAI when solving complex problems rather than relying on their logical reasoning and knowledge accumulation for analysis and resolution [35,39,40,50,55,69,70,72,74,75,77,98,99,124,136,146-152].

Second is decreased creativity. When students use GAI tools like ChatGPT, they often receive writing suggestions that lack the creativity and depth of human-generated content. Thus, prolonged reliance on such tools may weaken their independent writing skills and hinder their ability to engage with complex topics that require critical thinking and practical expertise [28]. Similarly, educators who overly depend on GAI for content creation may stifle their curricular innovation, limit diversity and depth in teaching materials, and ultimately diminish the overall quality of education [60].

Third is decreased teamwork ability. Overreliance on GAI tools such as ChatGPT can weaken students’ communication skills and ability to engage actively in collaborative teamwork [72,152]. Furthermore, the frequent use of these tools limits opportunities for meaningful interpersonal interaction with peers and mentors, hindering the development of essential teamwork and communication skills [152].

Fourth is decreased practical problem-solving ability. Practical problem-solving is essential for clinical decision-making and patient management. However, the convenience of GAI tools may lead students to rely on preexisting solutions, neglecting the deeper analysis and logical reasoning necessary to develop personalized answers [52,55,74,75,77,87,151-153]. Furthermore, using these tools may reduce interaction with mentors and peers, limiting students’ opportunities to gain diverse perspectives through collaborative discussions and approach problems from multiple angles [147].

#### Ethical Controversy

Ethical controversies can occur due to the following factors.

First is the authenticity of the test results, as the integration of GAI in testing and assessment may compromise the accuracy and effectiveness of traditional methods used to evaluate students’ actual capabilities. GAI-generated responses or GAI-assisted evaluations risk reflecting the performance of GAI itself rather than students’ authentic abilities. This issue is evident in various exams, such as medical licensing and specialty exams, presenting new ethical challenges in medical education [27,50,78,99,144,146,154].

Second is academic misconduct, since GAI-generated content often evades traditional plagiarism detection tools, making it easier for students to exploit GAI tools to complete assignments or write papers without being detected, thus jeopardizing academic integrity [31,154]. In addition, the ease of using GAI to generate answers cultivates students’ mindset of overreliance on such tools for academic tasks, which may increase their future likelihood of academic misconduct [70-72,124,155]. This issue extends beyond individual students and poses a broader threat to academic ethics, as GAI-generated content can be misinterpreted as original work, distorting academic evaluations [125,150].

Third is a lack of clinical interaction and emotional resonance. When addressing complex ethical or emotional medical issues, GAI lacks the empathy and emotional responsiveness inherent in human physicians, potentially undermining trust in the doctor-patient relationship [98,131]. This limitation is supported by a General Medicine In-Training Examination (GM-ITE) study comparing GPT-4 and Japanese residents. In the GM-ITE, “medical interview and professionalism” category assesses patient communication, ethics, and professionalism. It uses scenario-based questions (eg, addressing a terminally ill patient’s anxiety or resolving treatment ethics). Responses are scored 0‐10 based on communication appropriateness, empathy depth, and ethical application, with top marks for nuanced, human-centric judgment. Notably, GPT-4 scored 8.6 points lower here than residents [38]. Furthermore, because GAI tools do not provide an authentic, interactive experience or situational awareness, they may struggle to simulate the behavior and reactions of real patients accurately. This limitation makes it challenging for students to fully appreciate the importance of empathy and its application in doctor-patient interactions, which affects their development of communication and empathy skills development [38,54,76,77,101,107,147,152].

Fourth is resource inequality, which is most evident in the unequal access to technology and data. Datasets used for training GAI often exhibit biases, particularly involving data from different racial or socioeconomic backgrounds. This can worsen existing health care disparities. Furthermore, developing high-quality LLMs requires substantial computational resources, creating significant access barriers, especially for educational institutions or students with limited financial means. Hence, subscription fees and hardware limitations restrict their access to these GAI tools [67,74,85,134].

Fifth is the ownership of intellectual property rights. The widespread use of GAI in medical education raises numerous intellectual property concerns, particularly regarding copyright disputes related to the medical data used during AI training [113]. In addition, the legal status of GAI-generated content remains unclear, as current copyright laws do not adequately address the ownership of GAI-generated images and texts. This leaves the ownership of such content unclear, complicating the determination of whether the rights belong to the user, the developer, or other stakeholders [27,50,58,59,124].

Sixth is the “black box” problem and the attribution of responsibility. The application of GAI in medical education faces a significant challenge known as the “black box” problem. This issue arises from the lack of transparency and interpretability of GAI models, which directly affects the safety and reliability of these applications in medical settings. This lack of transparency makes it hard to understand how GAI reaches specific conclusions, especially when results are erroneous or biased, complicating efforts to trace and correct mistakes [86,88,148]. Furthermore, when GAI is used for diagnostic or clinical decision support, any errors or biases in its generated results can make it difficult to establish accountability. Trust in the doctor-patient relationship is built on clear responsibility. However, the lack of transparency in GAI models undermines this trust, leaving patients and physicians uncertain about the safety and reliability of GAI-driven decisions [36,114].

## Discussion

### Principal Findings

This scoping review systematically identifies 3 core characteristics of GAI in medical education through an analysis of 131 included studies: pronounced regional disparities, empowerment potential via RMA synergy, and unresolved technical and ethical challenges. These findings must be contextualized within the field’s evolving landscape: Our initial screening retrieved 5991 articles, a striking number reflecting both the opportunities and challenges of this emerging domain. This vast volume can be attributed to GAI’s rapid evolution as a nascent technology, where relevant concepts remain loosely defined and inconsistent. Consequently, keyword usage lacks standardization, often resulting in the inclusion of tangentially related cross-disciplinary studies. Furthermore, GAI’s inherently interdisciplinary nature broadens the scope of relevant literature. While this abundance highlights widespread interest and diverse applications, it also emphasizes the lack of conceptual clarity and consistency in frameworks. Therefore, although research is progressing, the field remains in a transitional stage, moving from “conceptual standardization” to “unified frameworks.” To propel the field forward, the academic community needs to reach a consensus on GAI-related definitions and application structures. Achieving this standardization will enable better tracking of emerging trends and facilitate the effective use of new insights.

Against this backdrop, regional distribution analysis reveals marked concentration of GAI research in very high HDI regions (74%), with minimal contributions from low-HDI regions (2%) and scarce cross-regional collaborations (4%), highlighting structural inequities in global technology diffusion. Model use patterns further demonstrate ChatGPT’s dominant adoption (89.5%), driven by its superior performance in multifaceted educational tasks: (1) iterative version advancements (eg, GPT-4’s significant improvements in reasoning accuracy and error reduction over GPT-3.5); (2) proven efficacy across diverse applications including clinical simulation, exam question generation, and personalized tutoring; and (3) robust multilingual support despite variability in non-English contexts. This technical versatility explains its preferential adoption by researchers. The disproportionately high usage rate of general LLMs over specialized models, coupled with a predominant focus on cross-model comparisons rather than synergistic integration, reflects insufficient exploration of technical adaptability and system interoperability within current research.

Within the RMA tripartite framework established in this study, GAI reshapes medical education through coordinated optimization across 3 dimensions. In resource provisioning, it effectively mitigates traditional constraints of specimen scarcity and privacy limitations through the efficient generation of diverse clinical cases and pathological images. Methodologically, it facilitates the transition from standardized instruction to personalized education through interdisciplinary knowledge integration and targeted learning support. For assessment, high concordance in automated scoring and academic integrity monitoring provides scalable solutions for educational quality assurance. This closed-loop optimization mechanism, which encompasses resource allocation, pedagogical implementation, and evaluative feedback, validates the framework’s explanatory power for technology-enabled educational transformation.

Nevertheless, profound barriers impede deeper GAI integration. Current technical deficiencies manifest as: inadequate contextual adaptation (eg, limitations in complex clinical reasoning and MCQ processing), data quality flaws (including hallucinatory outputs and deficient nontextual information analysis), and linguistic or regional biases (particularly performance degradation in non-English contexts). Long-term risks include erosion of critical thinking and creativity due to overreliance, alongside ethical governance dilemmas that encompass ambiguous accountability, inequitable resource distribution, and deficient clinician-patient emotional engagement. These dual challenges constitute fundamental barriers to implementing human-AI collaboration paradigms.

### Comparison With Existing Literature

This scoping review specifically focuses on the period between January 2023 and October 2024, a critical transitional phase where GAI in medical education shifted from theoretical exploration to practical implementation. By capturing this transformative era, it addresses the gap in previous reviews [1,9] that lacked coverage of the latest advancements. While building on the foundational insights of earlier studies, this review extends their scope by identifying emerging trends and practical applications that have emerged with GAI’s maturation in educational contexts.

Our observation of pronounced regional disparities starkly aligns with and quantifies the well-documented “digital divide” prevalent in global health technology diffusion [156]. However, this study provides concrete, GAI-specific evidence within medical education, highlighting the extreme concentration and the critical scarcity of cross-tier collaboration, thereby reinforcing concerns about equity in accessing transformative educational technologies and potentially exacerbating global health workforce inequities.

Regarding model use, the overwhelming dominance of ChatGPT mirrors its widespread popularity in GAI application studies [157]. Yet, our analysis delves deeper than mere prevalence reports or bibliometric study [158-160], specifically attributing this dominance to its rapid iteration (eg, GPT-4’s improvements), proven versatility across key educational tasks (clinical sim, QG, and tutoring), and relatively robust (though imperfect) multilingual support, which are crucial for adoption in the diverse contexts of medical education research.

Our development of the RMA tripartite framework represents a key theoretical departure. While existing research acknowledges GAI’s impact on discrete educational facets (resource provision, teaching methodologies, and evaluative processes), a unifying framework that binds these elements into a synergistic, closed-loop optimization mechanism is conspicuously absent from the current discourse [1,9,10]. Such a framework uniquely conceptualizes these three dimensions as an interdependent, dynamic closed-loop system essential for understanding GAI’s holistic transformative potential. Crucially, the empirical identification of significant RMA imbalance (robust exploration of educational methods and resources vs sparse focus on learner assessment) does not imply that assessment is under-prioritized in education broadly, but rather reflects a current skew in GAI-medical education integration—with research disproportionately focusing on resource enrichment and methodological optimization, while lagging in the development of learner assessment applications [161]. This imbalance, viewed through our novel integrative lens, offers a structured diagnostic for the systemic gap in aligning GAI capabilities with the specific needs of learner assessment within medical education.

The unresolved technical-ethical challenges documented (eg, contextual limitations, hallucinations, biases, erosion of critical thinking, and concerns about empathy) resonate strongly with growing critiques of LLMs in healthcare [162,163]. Our review explicitly maps these well-recognized limitations onto the sensitive context of medical education, highlighting their manifestation and potential impact in shaping future clinicians. This reinforces concerns raised elsewhere but grounds them firmly in the educational domain.

Another distinctive contribution of this review lies in revealing a critical technological imbalance: the overwhelming focus on general-purpose LLMs like ChatGPT contrasts sharply with the lack of systematic development of specialized medical models and the near absence of research on multimodel collaborative mechanisms within medical education [10,164]. This finding highlights a gap in the current technological approach, which hinders depth and clinical authenticity. While previous studies used available tools, our synthesis highlights this specific limitation as a barrier to deeper integration.

### Implications of the Findings

#### Implications for Educational Practice

This study makes a key contribution to pedagogical practice by establishing the RMA tripartite framework and revealing its developmental imbalances, thereby providing a practical paradigm for the integration of GAI into medical education. The core value of this framework lies in elucidating the dynamic closed-loop nature of technology-enhanced education, wherein resource provision establishes the pedagogical foundation, methodological innovation activates knowledge transformation, and assessment feedback drives systemic evolution; these 3 components constitute an interlocking educational mechanism [165].

As evidenced in the results section, the current imbalance, characterized by rich exploration in GAI-supported educational resources and teaching methods yet relatively limited progress in GAI-driven automated evaluation of learner performance, stems from an overemphasis on short-term efficiency in early technology adoption. This has led to systemic neglect of assessment’s role as an optimization tool. For example, GAI is widely used to generate diverse clinical cases and pathological images to enrich educational resources and design adaptive learning pathways to innovate teaching methods. However, in learner assessment, most GAI tools still rely on simple automated scoring of knowledge-based quizzes, with few leveraging GAI to evaluate higher-order competencies such as clinical reasoning or diagnostic accuracy [26]. Another instance is that many researchers use GAI to create interactive simulation scenarios as a methodological advancement but fail to integrate automated assessment features that track learners’ decision-making processes in these scenarios [53]. This misses opportunities to use assessment data to refine the scenarios themselves. Overreliance on GAI for resource and method innovation without matching progress in automated learner assessment risks disconnecting what is taught or provided from what learners need to master, ultimately limiting GAI’s ability to drive meaningful change in medical education.

Achieving optimal integration requires establishing a bidirectional enhancement cycle centered on assessment. Automated assessment data capturing learning bottlenecks should guide the real-time expansion of clinical case libraries’ pathological spectra and difficulty calibration [166], shifting resource provision from one-size-fits-all to demand responsiveness. Simultaneously, the focus on core competencies (such as clinical reasoning and problem-solving) emphasized in teaching methods must be integrated into new assessment dimensions [167], driving teaching methods to evolve from mere knowledge transmission to competency development. Within this cycle, assessment functions not merely as a quality monitoring tool, but as the central nexus for the co-evolution of resources and methods.

Realizing this vision necessitates educators reconceptualizing operational logic [165]. This involves using assessment data to inform the development of educational resources, specifically leveraging insights into learners’ knowledge gaps and skill deficiencies to dynamically adjust the complexity of clinical cases [168], embedding real-time, practical, and contextual feedback mechanisms within high-order teaching activities like simulated diagnostics to optimize pedagogical strategies [169], and establishing adaptive rules enabling cross-dimensional interaction to facilitate systemic iteration [170,171]. Collectively, this structural transformation elevates the tripartite framework into an organic educational operating system.

However, technological integration inherently presents dual challenges, highlighting the importance of upholding core principles of human-AI collaboration. Generating educational resources without clinical context review risks reinforcing data biases [172]; methodological innovation overly reliant on algorithmic decisions may erode critical thinking [9]; and automated assessment replacing human judgment may overlook students’ psychological needs, reducing course engagement and well-being scores [173]. These manifestations of technological alienation arise from the partial ceding of human agency. Resolution lies in upholding a human-AI symbiotic vision: recognizing GAI as a collaborator, not a replacement, in educational evolution. Specifically at the resource layer, clinicians and educators must oversee the development of educational resources (eg, clinical cases) to balance efficiency, ethics, and clinical authenticity [174,175]. At the method layer, educators should direct learning path design to integrate technological augmentation with pedagogical wisdom [176]. At the assessment layer, institutions should implement verification systems that combine human evaluation with machine automation, ensuring assessments balance efficiency with humanistic dimensions [173,177]. This reconfiguration of responsibilities positions technology as a tool and reaffirms human stewardship of education.

#### Implications for Technological Development

This study identifies a technological imbalance in the application of GAI within medical education. This imbalance is characterized by the dominance of large general language models, while the development of specialized models for specific medical disciplines has lacked systematic progress. This limitation restricts the depth of technology-enabled education and indicates a neglect of multimodel collaborative mechanisms within current research paradigms.

The study proposes an integrated system using general LLMs alongside specialized medical models, employing a hierarchical collaborative architecture to reshape the technological ecosystem of medical education. The core operational logic establishes a 3-tiered functional division: general models act as the central hub for teaching interactions, handling basic task parsing and process orchestration; medical specialized models, drawing on vertical domain knowledge bases, execute high-complexity core teaching tasks such as clinical reasoning and medical image generation; and a cross-model validation mechanism forms a closed-loop quality control system. This architecture adapts the hospital’s multidisciplinary team approach to AI in education, aligning technological capabilities with the requirements of medical education for expertise, reliability, and contextual authenticity.

Within medical education, this integrated system can facilitate 3 key changes. First, it addresses limitations in specialized knowledge depth inherent in traditional general models, improving training efficacy for advanced clinical reasoning. Second, it leverages GAI’s multimodal capabilities, which integrate text and image data, to address key issues in medical imaging education including shortages of teaching resources like rare pathological images and the limits of static materials in showing dynamic anatomical relationships. This support helps evolve pathology visualization from static atlases to interactive 3D simulations, letting students explore spatial structures and pathological changes more intuitively [178]. Third, it establishes a cross-model knowledge validation chain to automatically identify and correct typical logical inconsistencies and factual errors in general models, ensuring the academic rigor of teaching content. These changes collectively represent a paradigm shift from tool-assisted learning to intelligent teaching partnership systems [179].

Supporting the effective operation of this system requires targeted solutions to key technical challenges. The primary task involves developing specialized models with medical context adaptive capabilities, specifically enhancing their semantic parsing of unstructured clinical texts to address performance variability in complex case analysis [180,181]. Concurrently, it is necessary to construct dynamically evolving medical education datasets that incorporate cross-regional case spectra and multilingual clinical literature to systematically mitigate cultural biases and time-lag effects in training data [182]. Integrating privacy-preserving computation techniques like federated learning can enable secure data collaboration among institutions, continuously optimizing model localization and adaptation while safeguarding patient information security [183,184].

#### Implications for Policies and Governance

This study reveals a pronounced regional disparity in the application of GAI within the field of medical education. Specifically, regions with a very high HDI dominate research output in this domain, while contributions from the low-HDI areas account for only 2%. The scarcity of cross-tier collaboration between very high- and low-HDI areas further exacerbates this structural inequity in resource distribution. This imbalance epitomizes systemic inequalities within global knowledge production systems, rooted in 3 compounding barriers: inadequate computational infrastructure in resource-constrained settings impedes technological localization, proprietary restrictions on core models under patent regimes limit feasible technology transfer, and excessive reliance on clinical data from high-income countries compromises model adaptability to regional health care priorities. Without deliberate intervention, this self-reinforcing Matthew Effect cycle risks intensifying the global fragmentation of medical educational resources [185].

Addressing this complex challenge necessitates a multitiered governance framework. At the international level, binding technology-sharing agreements should request that holders of advanced models provide architectural access under fair-use principles, emphasizing the need to balance innovation with equitable access, while emulating open-source paradigms as a reference model [186]. Concurrently, the World Health Organization could coordinate multinational efforts to develop nonprofit medical corpora incorporating disease spectra prevalent in low-HDI regions, such as tropical and endemic diseases [187]. Nationally, ministries of education should integrate computational infrastructure into public medical education budgets [188] and similar to the Medical Education Partnership Initiative (MEPI) [189], establish dedicated funds for cross-border institutional partnerships to co-develop localized pedagogical tools that address specific regional educational needs. Institutionally, medical schools should adopt algorithmic transparency protocols, requiring deployed GAI tools to provide auditable model documentation that details the demographics and geographical coverage of training data. Fairness assessments of these tools should be carried out by multidisciplinary committees, which include clinicians, ethicists, and community representatives [190].

Simultaneously, institutional responses must address secondary risks through integrated technical, educational, and regulatory safeguards. To counter academic misconduct, educational institutions should implement dual-track verification systems that require GAI-assisted submissions to be accompanied by generation logs and validated through detection tools [191]. Academic journals must establish clear authorship standards declaring proportional human-GAI contributions [192]. Mitigating critical thinking erosion requires curriculum committees to incorporate GAI-free clinical reasoning assessments, such as on-site case analyses evaluating independent diagnostic and management planning capabilities as prerequisites for professional certification [193].

Technical deficiencies demand targeted interventions. Reducing model hallucinations requires dynamic fact-checking systems linking GAI outputs to authoritative medical knowledge bases, with confidence levels displayed during teaching platform usage [194]. To address the opacity of algorithms, where the process by which GAI models derive conclusions remains unclear, it is necessary to document the diagnostic reasoning processes of these models. Such documentation allows instructors to review the reasoning, helps determine accountability when inconsistencies occur, and can be integrated into resident training evaluations to strengthen oversight of GAI-assisted decision-making [195].

**Figure 5.**
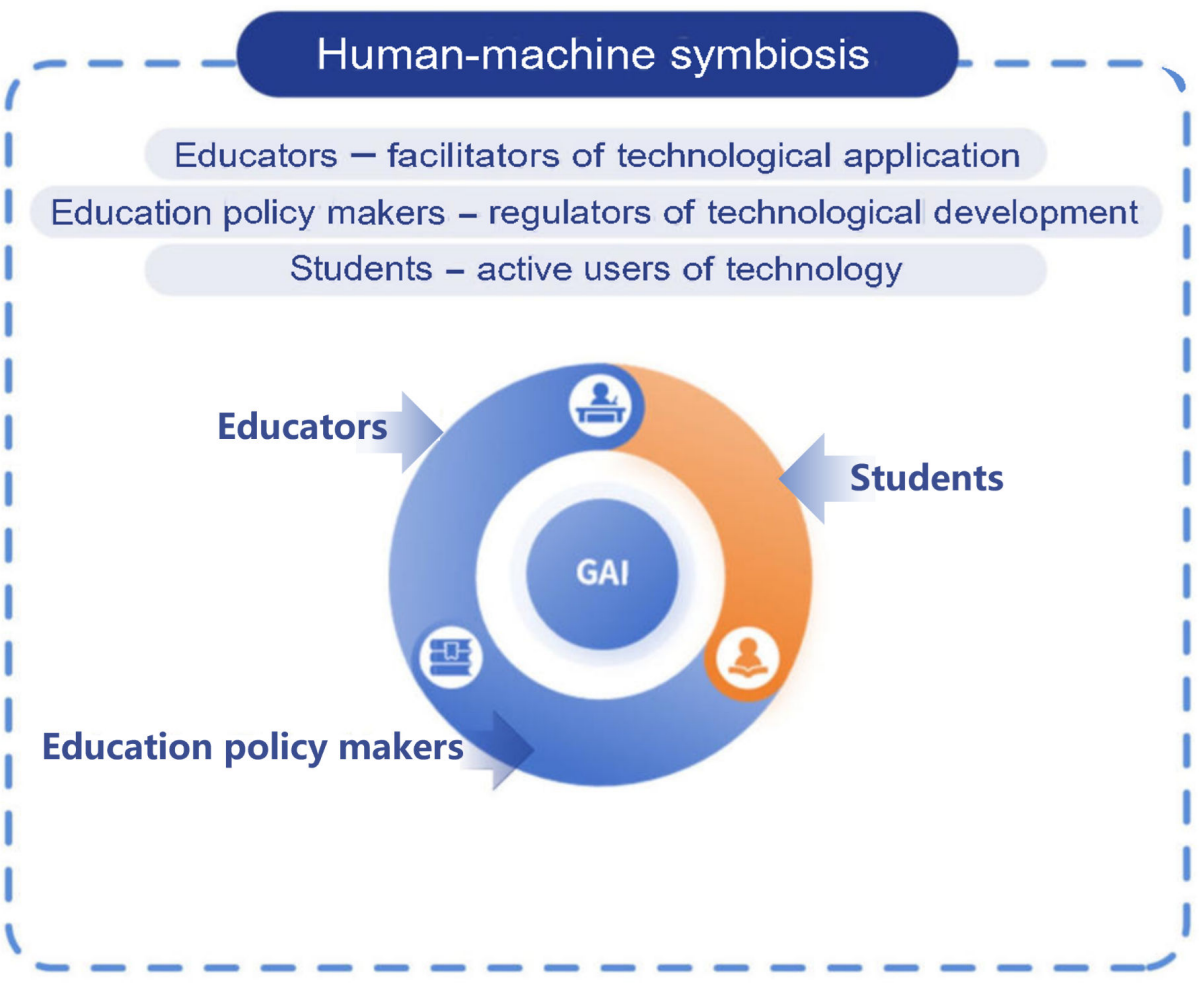
Vision of human-machine symbiosis: a schematic diagram. GAI: generative artificial intelligence.

Fundamentally, governance paradigms must transition from a technocentric approach to symbiotic development. Compared to the commonly used “human in the loop” [196], which mainly emphasizes humans overseeing or making final decisions in AI systems, symbiotic agency theory goes further: it highlights mutual shaping between humans and AI. Humans guide AI development through ethical norms and clinical experience, while AI enhances human capabilities by expanding cognitive boundaries, forming a dynamic, mutually reinforcing relationship [11]. Policies should affirm human primacy in medical education, exemplified by reserving clinical empathy training exclusively for human instructors while limiting GAI to standardized case supplementation. An effective return to the essence of symbiotic agency means building collaborative mechanisms as shown in Figure 5: educators lead in setting teaching goals and ensuring ethical alignment (eg, reviewing GAI-generated cases to match real clinical logic); GAI supports personalized learning; students provide feedback to refine GAI tools; and policies clarify rights and responsibilities in this interaction. This human-centered approach ensures technological advancement aligns with pedagogical integrity and global equity imperatives.

### Limitations and Future Direction

This scoping review has several limitations that should be acknowledged. First, the rapidly evolving nature of GAI means our findings primarily reflect the landscape captured up to the search date; newer models and applications emerging subsequently may shift current patterns. Second, the inherent conceptual breadth and interdisciplinary nature of GAI pose challenges for exhaustive literature capture, potentially leading to omissions despite broad search parameters. Third, and most critically, while this study proposes 3 key conceptual frameworks (the RMA tripartite model, the hierarchical collaborative architecture, and the symbiotic agency principle) and argues for their feasibility based on synthesized evidence, it has not empirically tested their implementation or efficacy in authentic educational settings. Finally, reliance on published literature may underrepresent real-world implementation challenges and grassroots innovations.

Future research must bridge this critical gap by translating these frameworks into practice. Priority should be given to: (1) implementing and evaluating the RMA balancing strategies and the integrated system combining general and specialized medical GAI models in specific medical education contexts to assess their impact on learning outcomes and operational feasibility; (2) conducting longitudinal studies to track the dynamic evolution of GAI integration over time, observing its long-term empowerment effects on educational processes and outcomes; and (3) operationalizing the symbiotic agency framework to guide the design, deployment, and assessment of these interventions. This framework is essential for ensuring that human-AI collaboration in practice genuinely augments educator and learner agency, fosters critical competencies, and upholds pedagogical integrity, thereby realizing the envisioned synergistic educational ecosystem.

### Conclusion

The application of GAI in medical education exhibits significant regional inequities, reflecting structural disparities in technological diffusion. Statistical findings from the model research reflect that researchers have certain preferences in its usage. The emergence of GAI has revitalized medical education, which is manifested in its promotion of the diversification of educational methods, the scientific evaluation of education assessment, and the dynamic optimization of education resources. However, these innovations are accompanied by current limitations and potential future challenges. By establishing the RMA tripartite model as a dynamic closed-loop system for educational optimization, proposing an integrated multimodel architecture to reconcile general and specialized GAI capabilities, and advancing the symbiotic agency principle to safeguard human primacy, this study provides foundational frameworks for navigating GAI integration. These contributions collectively address critical gaps in conceptual standardization and collaborative design, while delineating actionable pathways for pedagogical innovation, equitable technology development, and governance reform, which ultimately steer the field toward responsible human-AI collaboration that enhances clinical education without compromising pedagogical integrity or global equity.

## Supplementary material

10.2196/71125 Multimedia Appendix 1 Technical features and application comparison of mainstream generative artificial intelligence (GAI) models.

10.2196/71125 Multimedia Appendix 2 Search strategy.

10.2196/71125 Checklist 1 PRISMA-ScR checklist.

## Abbreviations

AI: artificial intelligence
GAI: generative artificial intelligence
HDI: Human Development Index
LLM: large language model
MCQ: multiple-choice question
RMA: resource-method-assessment
SAQ: short-answer question
