# Supplementary material for: Applications, Challenges, and Prospects of Generative Artificial Intelligence Empowering Medical Education: Scoping Review
Source: JMIR Med Educ. 2025 Oct 23;11:e71125. doi: 10.2196/71125 (PMC12547994; doi:10.2196/71125)
Supplement: Multimedia Appendix 1 [file mededu-v11-e71125-s001.docx]

Table S1 **Technical Features and Application Comparison of Mainstream GAI Models**

| Company | Model | Technical Features | Core Capabilities | Optimal Application Scenarios | Key Limitations |
| --- | --- | --- | --- | --- | --- |
| Open Al | O3 | Multimodal input (text/images); depth reasoning architecture optimization; dynamic tool orchestration framework; expanded token context window; advanced safety alignment mechanisms | Mathematical proof generation, code debugging, and complex scientific hypothesis verification; self-fact-checking with iterative reasoning loops | Professional scenario analysis (e.g., financial risk modeling, legal document interpretation); scientific hypothesis generation | Higher hallucination rate (33% in PersonQA benchmark) in interdisciplinary tasks; high computational resource demand |
| Open Al | GPT-4 | Large token context window; multimodal fusion (text/image understanding); iterative self-correction mechanisms | Complex task orchestration (e.g., literature review synthesis, cross-disciplinary report writing); nuanced semantic comprehension | General academic research support; comprehensive content creation | Controversial reasoning capabilities (preprint studies question logical consistency); occasional factual inaccuracies in specialized domains |
| Microsoft | Copilot  (originally Bing） | Windows ecosystem integration; real-time collaborative editing; low-latency response | Instant code completion; document formatting optimization; multilingual programming support | Software development (e.g., real-time code debugging, cross-language project management); daily office automation | Limited domain specialization; |
| Google | Gemini (originally Bard) | Multilingual parallel processing (multiple languages with native audio output); cross-modal reasoning (text→code→image); hardware-accelerated performance | High-accuracy translation (technical terminology preservation); dynamic tone adaptation for multilingual technical documentation | Technical whitepaper analysis; cross-lingual software development; complex mathematical modeling | Suboptimal performance in low-resource language contexts |
| Meta | LLaMA 3 | mixture-of-experts (MoE) architecture for scalable customization; efficient fine-tuning on consumer-grade hardware | Custom model development; localized data training; algorithmic transparency research | Academic AI model R&D; institution-specific application customization (e.g., educational platform development) | Lower performance than closed-source models in high-complexity tasks |
| Anthropic | Claude 2 | Constitutional AI alignment framework; large token context window; audit trail logging | Ethical risk mitigation (bias detection in financial analysis); transparent reasoning process output | Scenarios requiring strict compliance (e.g., legal contract analysis, financial compliance auditing) | Slower response speed in high-complexity tasks |
| Baidu | ERNIE 4.0 | Chinese semantic parsing with high accuracy; multimodal fusion (text/image/video) | Chinese legal document generation; financial data analysis; policy document interpretation | Chinese-language legal services; domestic financial risk management; public policy research | Limited performance in non-Chinese contexts |
| Alibaba | Qwen 2 | Large token context window; cost-efficient inference; batch processing optimization | Large-scale content generation (textbooks, training manuals); enterprise-level knowledge base construction | Massive open online course (MOOC) development; corporate training material production | Less advanced in cross-lingual reasoning |
| OpenAI | Sora | Text-to-video generation (up to 20 seconds); dynamic scene continuity; advanced editing tools (remix, storyboard) | manufacturing workflow visualization; educational video generation | Product demonstration animation; industrial training module production; technical tutorial creation | High computational resource demand; challenges in complex 3D scene generation；Text-to-video generation may still be in its infancy |
| OpenAI | DALL·E 3 | High-resolution image output; precise inpainting; style consistency control | Scientific visualization (e.g., data chart generation); anatomical structure illustration | Academic publication illustration; educational infographic design; basic scientific diagram creation | Inconsistent accuracy in rare disease and complex medical image generation |
| Adobe | Adobe Firefly 1.0 | Creative Cloud integration; copyright-compliant training data; layer-based editing support | Clinical diagram customization (e.g., organ system labeling); educational poster design | Graphic design workflow optimization; enterprise-level visual content production | less specialized in clinical imagery |
| OpenEvidence | OpenEvidence | Domain-specific training (Cochrane Library, PubMed corpus); systematic review automation; compliance checking with NEJM/JAMA integration | Evidence-based medical recommendation; clinical trial data synthesis | Medical education (evidence-based practice training); clinical decision support | Narrow application scope (non-medical scenarios excluded) |
