# Supplementary material for: Applications, Challenges, and Prospects of Generative Artificial Intelligence Empowering Medical Education: Scoping Review
Source: JMIR Med Educ. 2025 Oct 23;11:e71125. doi: 10.2196/71125 (PMC12547994; doi:10.2196/71125)
Supplement: Multimedia Appendix 2 [file mededu-v11-e71125-s002.doc]

1.PubMed

(("Artificial Intelligence"[Mesh] OR "Generative AI"[All Fields] OR "Generative Models"[All Fields] OR "Large Language Models"[All Fields] OR "GPT"[All Fields] OR "Copilot"[All Fields]) OR ("Artificial Intelligence"[Mesh] OR "Generative AI"[All Fields] OR "Generative Models"[All Fields] OR "Large Language Models"[All Fields] OR "GPT"[All Fields] OR "Copilot"[All Fields]) OR (("Artificial Intelligence"[Mesh] OR "Generative AI"[All Fields] OR "Generative Model"[All Fields] OR "Large Language Models"[All Fields] OR "GPT"[All Fields] OR "Copilot"[All Fields]) OR "Artificial Intelligence"[Mesh] OR "Generative Models"[All Fields] OR "Generative AI"[All Fields] OR "large language models"[All Fields] OR "gpt"[All Fields] OR "Copilot"[All Fields])) AND ((("Education, Medical"[Mesh] OR "Medical Education"[All Fields] OR "Clinical Education"[All Fields] OR "Teaching"OR "medical learning"[All Fields]) OR (e-learning[All Fields] OR "blended learning"[All Fields])) AND ((fft[Filter]) AND (english[Filter]) AND (2023:2024[pdat])))

2.Web of Science

(TS=("Artificial Intelligence" OR "Generative AI" OR "Large Language Models" OR "Generative Models" OR "GPT" OR "Copilot")) AND TS=("Education, Medical" OR "Medical Education" OR "Clinical Education" OR "Teaching" OR "medical learning" OR e-learning OR "Blended Learning")


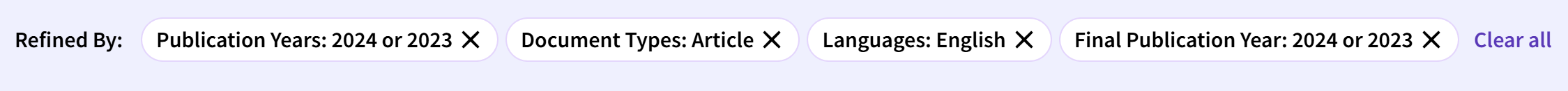


3.Scopus

TITLE-ABS-KEY ( "Artificial Intelligence" OR "Generative AI" OR "Large Language Models" OR "Generative Models" OR "GPT" OR "Copilot" ) AND TITLE-ABS-KEY ( "Education, Medical" OR "Medical Education" OR "Clinical Education" OR "Teaching" OR "medical learning" OR e-learning OR "Blended Learning" ) AND PUBYEAR > 2022 AND PUBYEAR < 2025 AND PUBYEAR > 2022 AND PUBYEAR < 2025 AND PUBYEAR > 2022 AND PUBYEAR < 2025 AND ( LIMIT-TO ( DOCTYPE , "ar" ) OR LIMIT-TO ( DOCTYPE , "Systematic Review" ) OR LIMIT-TO ( DOCTYPE , "Meta Analysis" ) ) AND ( LIMIT-TO ( LANGUAGE , "English" ) )
